# Measuring What Counts in Life: The Development and Initial Validation of the Fulfilled Life Scale (FLS)

**DOI:** 10.3389/fpsyg.2021.795931

**Published:** 2022-01-11

**Authors:** Doris Baumann, Willibald Ruch

**Affiliations:** Department of Psychology, University of Zürich, Zurich, Switzerland

**Keywords:** positive psychological assessment, wellbeing assessment, scale development, validation, positive psychology, life span, positive aging, fulfilled life

## Abstract

In a recent work, we introduced a theoretical model for fulfillment in life that covers cognitive and affective components and distinguishes different time frames. The present study evaluates this model and describes the construction of the Fulfilled Life Scale (FLS) to assess fulfillment regarding the whole lived life retrospectively. We investigated the scale in two samples (Sample 1: *N* = 282 adults aged 50–93 years; Sample 2: *N* = 406 adults aged 40–85 years). The model of the cognitive component combines three sources of fulfillment (*self, life, impact/legacy*) with three criteria (*wholeness, fit, value*), yielding nine facets. Employing hierarchical factor analysis, we inspected all solutions between one and nine. We identified three optimal factors, which we labeled *unfolded self and life*, *the worthwhile life*, and *positive impact and legacy*. Next, we selected marker items and replicated the factor structure in Sample 2. The three scales were positively intercorrelated and showed good internal consistency in both samples. For the affective component, exploratory and confirmatory factor analyses established a one-factor structure in both samples, and high internal consistency was obtained. Across a range of related constructs, we demonstrated construct and criterion validity. Notably, cognitive and affective fulfillment incrementally predicted a global rating of a fulfilled life and mental well-being, even after controlling for subjective and eudaimonic well-being. Overall, the study proves that the FLS is necessary to capture people’s experience of a fulfilled life, which could not be assessed sufficiently with previous well-being measures. Both cognitive and affective fulfillment were able to predict additional variance in mental well-being. Moreover, the study reveals psychometric support for the FLS and presents the first evidence on its validity. Lastly, applications in research and practice are discussed, especially in the context of living and aging well in the second half of life.

## Introduction

If psychologists wish to improve the human condition, it is not enough to help those who suffer. The majority of “normal” people also need examples and advice to reach a richer and more fulfilling existence (Seligman and Csikszentmihalyi, 2000, p. 10).

From the inception of the field, positive psychology has emphasized fulfillment in life (FiL) as a central topic. Though the term has appeared regularly in the literature, virtually no related research has taken place. This gap in the research can be attributed to the lack of a theoretical conceptualization and the absence of an instrument for its assessment (Baumann and Ruch, 2021). Consequently, we proposed a definition along with a theoretical model for FiL that distinguishes several time frames, from fulfillment in an activity to perceiving one’s life as fulfilled. The present study serves as a next step, having the objective to test this model and develop a measure that will assess a fulfilled life. We decided to focus on a fulfilled life in retrospect, as taking the whole lived life into account for an evaluation seems to have the greatest relevance, more than a fulfilling activity or having had a fulfilling life at a particular life stage. What counts is that individuals can look back and arrive at the conclusion that their lives were fulfilled. The availability of such a scale and the resulting findings will contribute to a deeper understanding of the construct, facilitate building and expanding its nomological network, and may further stimulate research on FiL. In turn, insights into a fulfilled life may have important implications for practice. Because life satisfaction does not seem to be the sole criterion of how individuals evaluate their lives and how well they age (Westerhof et al., 2001), our scale measuring a fulfilled life can provide valuable insights from a different angle.

### Conceptualization of a Fulfilled Life

We defined FiL as “a cognitive-affective experience referring to a sense of wholeness, fit, and value toward the self, one’s life, and one’s impact” (Baumann and Ruch, 2021, p. 6). Accordingly, a fulfilled life refers to the positive appraisal of the person one has become, how one has led one’s life, and the impact one has made. The FiL model represents the cognitive component as a 3 × 3 matrix (see Table 1) that combines the three criteria for fulfillment *(wholeness, fit, and value)* with the three sources of fulfillment *(self, life, and impact/legacy)*. The sources constitute the three main strands from which individuals derive fulfillment when referring to life as a whole. The combination of the criteria and sources yields nine facets that are presented in the FiL model as outlined in Table 1: (a) *realized uniqueness*, (b) *a life lived fully*, (c) *the making of a positive difference*, (d) *authentic pursuits*, (e) *a life true to oneself*, (f) *a contribution reflecting the self*, (g) *worthwhile involvements*, (h) *a life that was worthwhile, and* (i) *a life that mattered to others*. Table 1 displays a brief description of the nine facets (for more details, see Baumann and Ruch, 2021).

**TABLE 1:** The fulfillment in life (FiL) model depicting the cognitive-evaluative component.

Criteria for fulfillment	Sources of fulfillment		
	Self	Life	Impact/Legacy
*Wholeness* Sense of wholeness and completeness	*(A) Realized Uniqueness:* Fulfillment from having been able to become more fully oneself	*(B) A Life Lived Fully:* Fulfillment from realizing life goals and having lived life consciously	*(C) The Making of a Positive Difference:* Fulfillment from having been able to make a positive contribution and to leave something of value
*Fit* Sense of congruence and alignment	*(D) Authentic Pursuits:* Fulfillment from having had the courage to be true to oneself	*(E) A Life True to Oneself:* Fulfillment from having led a life that felt right	*(F) A Contribution Reflecting the Self:* Fulfillment from having been able to combine own values, talents, and interests while making a positive contribution
*Value* Sense of meaningfulness, significance, worthwhileness	*(G) Worthwhile Involvements:* Fulfillment from having used one’s resources and potentialities sensibly	*(H) A Life that was Worthwhile:* Fulfillment from perceiving one’s life as worthwhile and meaningful	*(I) A Life that Mattered to others:* Fulfillment from a sense that one’s life mattered and made a positive difference to others

*Rows represent criteria for fulfillment, columns represent sources of fulfillment, and the cells represent the major content of the nine cognitive facets of a fulfilled life.*

This arrangement of criteria and sources provides a systematized way to represent prior thinking. The experience of fulfillment requires certain qualities (the criteria) to be present to a sufficient extent. As delineated in our theoretical article (Baumann and Ruch, 2021), the first criterion of wholeness designates the extent to which one could become a whole person, live life fully, and positively influence others’ lives. The criterion of fit refers to a sense of congruence and alignment and involves the perception that one was true to the self, lived a life that suited one deeply, and has been able to make a contribution that is reflective of what one holds dear. Value as a third qualitative requirement for fulfillment relates to the perception that one has invested one’s own capacities well and lived a worthwhile and meaningful life; moreover, one’s life will have had value and mattered to others. Affective fulfillment was considered to consists of low-arousal positive affect comprising such feelings as inner contentment, gratefulness, harmony with oneself and one’s life, or inner peace (Baumann and Ruch, 2021). In addition, a fulfilled life is characterized by the absence of intense negative affective experiences, such as feelings of emptiness, deep regret, or disappointment.

### Measuring a Fulfilled Life

A fulfilled life might be measured in different ways. Fulfillment is subjective in nature, which must be acknowledged in measurement. Even though developing a checklist of factors that are empirically confirmed to contribute to fulfillment is possible, the total score will not suffice unless each of the items is subjectively weighted. Peer evaluation might be hampered by a variety of biases but, at the same time, be useful as a validation criterion. A global subjective evaluation on an anchored rating scale might serve as a useful initial indicator, but there is no substitute for a genuine measurement. The anchoring may be established by having participants first describe their most apt example of a fulfilled life and then stipulate how close their own life is to their self-defined ideal. This latter approach is the one we chose for validation purposes.

Our preference has been to determine the contents of the evaluation by directly asking the questions that cover our model. Having economic measures for each of the nine facets from Table 1 as an intermediary state is certainly of interest, but most importantly is a measure of its essence (in other words, the factors underlying these facets). The results will reveal whether total scores for the cognitive and affective domains may be derived as well.

Another consideration relates to determining the best target audience for the scale being developed. We have set the target group to encompass middle-aged and older adults, as global life evaluations are more common with age (Westerhof et al., 2001); moreover, taking stock of one’s life requires a certain number of experiences. Some of what truly holds value or is significant might be recognizable only in retrospect, and what seems essential at one moment might lose its value at a later point in time. The development of the instrument reflects the following objectives: We determined that it should (a) be multidimensional to capture the nine cognitive facets and an affective component, (b) possess good psychometric properties, and (c) contain comprehensive content while also taking brevity into account. For scale construction, we have followed the standards as outlined by Simms (2008). The scale development process involved four phases (see Table 2): (a) developing items, employing expert review and cognitive pretesting; (b) evaluating the psychometric properties of the individual items, examining the factor structure, creating initial scales, and establishing reliability; (c) assessing factor structure, the similarity of factors, and the reliability of the final scale in a new sample; and (d) investigating convergent and discriminant validity and testing concurrent and incremental validity. This approach helped us address the content and structure of a fulfilled life, determine whether the proposition of a new construct and measure is justified, and identify how the construct is located in its nomological network.

**TABLE 2:** Phases of scale development.

Phases	Aims	Sample	Data analysis
Phase 1: Substantive validity	• Development of initial item pool		
	• Expert review		
	• Cognitive pretest		
Phase 2: Structural validity I	• Data collection in a sample of the target group	Sample 1	• Descriptive statistics
			• Hierarchical factor analysis
	• Psychometric evaluation of items		• Exploratory factor analysis
	• Item preselection		• Parallel analysis
	• Examination of factor structure		• Minimum average partial test
	• Creation of provisional scales		• Reliability coefficients
Phase 3: Structural validity II	• Assessment of factor structure	Sample 2	• Exploratory factor analysis
	• Test of similarity of factors		• Confirmatory factor analysis
	• Examination of reliability in a new sample		• Tucker’s Phi coefficients
			• Reliability coefficients
Phase 4: External validity	• Assessment of convergent validity	Sample 1 and 2	• Correlation analysis
	• Evaluation of discriminant validity	Sample 1	• Hierarchical regression analysis
	• Assessment of concurrent validity	Sample 1 and 2	
	• Assessment of incremental validity	Sample 1 and 2	• *t*-tests
	• Testing known groups validity	Sample 3	

### The Present Study

The main objective of this study involved developing and validating the Fulfilled Life Scale (FLS). In addition to a pilot form encompassing the nine facets, we intended to create an economical version, optimally comprising 20–30 items. Our pursuit of this aim began with an examination of the underlying factors. We expected that all cognitive facets would positively intercorrelate and that we would find a multi-dimensional factor structure beyond the global level. Our plan included establishing validity by investigating the relationship between a fulfilled life and similar constructs and the criteria it predicts, drawing on our theoretical article (Baumann and Ruch, 2021). In the first place, the participants’ global rating of how close one’s own life comes to a maximally fulfilled life serves as the prime validity criterion. A total score or components should highly correlate with a layperson’s view of fulfillment. Next, it is vital to show that fulfillment overlaps with concepts like life satisfaction, subjective well-being, and eudaimonic well-being without being redundant (i.e., containing additional unique variance). Two types of results will underscore the usefulness of fulfillment as a new concept. In one of these, reliable variance in the fulfillment measures should not be fully explained by the existing concepts alone or together (e.g., hedonic and eudaimonic well-being do not fully account for fulfillment). According to the other, fulfillment should exhibit incremental validity when predicting important life outcomes (i.e., should be predictive over and above traditional variables). In the present study, we explore whether affective and cognitive fulfillment can incrementally predict a global rating of a fulfilled life after controlling for established well-being measures, such as subjective and eudaimonic well-being. Furthermore, we suggest that a fulfilled life can predict relevant key variables for aging well, including prospective life satisfaction, mental well-being, and self-perceptions of aging. Establishing these relationships will be essential.

In particular, we expect that the orientations to engagement, meaning, and accomplishment relate to general fulfillment. Nevertheless, we also believe that positive relationships are more relevant in terms of leaving a legacy. We assume that a favorable psychosocial development should play a significant role in attaining a fulfilled life. Generativity and ego integrity are development tasks in the second half of life (Erikson, 1985), and their successful resolution leads to a mature, well-rounded personality. We expect both concepts to be related to a fulfilled life. As research has suggested that perceiving one’s job as a calling is fulfilling (Wrzesniewski et al., 1997; Hall and Chandler, 2005), we expect that persons arriving at a fulfilled life are more likely to report a calling. Furthermore, we intend to demonstrate known-groups validity by showing that selected individuals pursuing a calling (named calling exemplars) differ from a general sample regarding their level of fulfillment. Finally, we will report the results of comparisons with sociodemographic and contextual variables.

## Materials and Methods

### Phase 1: Substantive Validity

For the initial item pool generation, we used a rational-theoretical approach based on the theoretical model of FiL (Baumann and Ruch, 2021). The study of specialist and non-specialist literature about the different facets of a fulfilled life yielded rich sources for developing an item pool to ensure good content validity. Such an approach should ensure that the construct also occurs in everyday life and is wholly and accurately covered. We developed items to assess the nine cognitive facets and the affective component described in the introduction and illustrated in Table 1. The first author wrote 101 items to measure the cognitive component and 12 items for assessing the affective component. All statements referred to one’s life lived so far in retrospect. The cognitive items were positively worded, while the affective statements also comprised six negatively phrased items to be recoded. Items corresponding to the affective experience consisted of positive low-arousal feelings, such as deep inner contentment, inner peace, and negative feelings, including disappointment, emptiness, or deep regret when looking back on one’s life. The number of items was purposely overinclusive to be able to select those with the best psychometric properties and content coverage. We chose a 6-point Likert scale to ensure sufficient response variance and to avoid overwhelming older participants with a too large response format. Eight experts in the field of positive psychology (Ph.D. students, senior researchers, and a professor) who were also proficient with the concept of FiL, rated the content validity (the extent to which the items reflect the content domain) and item quality (comprehensibility, conciseness, and redundancy). We improved the wording of a few items, and selected the best nine items from each of the nine facets of the cognitive component. Construct validity was further improved by using a think-aloud procedure, a cognitive interviewing method, to identify and correct sources of response error in the survey questions. Employing this method in the early stage of test construction can prevent problems in the areas of comprehension, recall, or decision processes, enabling lay people to provide valuable information about the construct. After conducting three face-to-face cognitive interviews with persons from our target group, we modified the wording of four items. The provisional scale for further analysis comprised 81 cognitive and 12 affective items.

### Phase 2: Structural Validity I

#### Participants

Sample 1 (development sample) consisted of *N* = 282 German-speaking participants aged 50–93 (*M* = 60.93, *SD* = 8.64, 79.4% women). Two participants did not indicate their age. Of the sample, 50.7% were married or in a registered partnership, 25.2% were divorced, 15.2% were single and never married, 5.7% were widowed, and 3.2% were separated. Approximately half of the sample (53.2%) had attained a university degree as their highest level of education, while 20.6% held a professional education diploma, 11.7% had a general education (e.g., baccalaureate), 13.5% had a vocational education and training, and 1.1% had a compulsory school qualification (9 years of education).

Sample 2 (replication sample) included *N* = 406 German-speaking participants aged 40–85 (*M* = 58.81, *SD* = 10.66, 78.8% women). One participant did not indicate her age. Of this sample, 50.7% were married or in a registered partnership, 20.0% were single and never married, 20.9% were divorced, 5.7% were widowed, and 2.7% were separated. About half of the sample (49.5%) had attained a university degree as their highest level of education, while 25.1% held a professional education diploma, 12.3% had a general education (e.g., baccalaureate), 11.8% had a vocational education, and 1.2% had a compulsory school qualification (9 years of education).

Sample 3 (calling exemplars) consisted of *N* = 39 German-speaking participants aged 41–89 (*M* = 57.92; *SD* = 9.63; 79.5% men). Of these, 53.8% were married, 28.2% were divorced, 12.8 were single or never married, and 5.1% were widowed. This sample was highly educated: 71.8% held a university degree, 23.1% held a professional education diploma, 2.6% held a baccalaureate, and 2.6% had a compulsory school qualification.

The three samples included only participants who provided complete and valid responses. Participants were excluded when they did not meet the inclusion criteria or showed obvious or irregular response patterns (Sample 1: *n* = 6; Sample 2: *n* = 13; Sample 3: *n* = 4). Other outliers were kept and were in the range of what is expected in a normal distribution.

#### Procedure

We collected data for all samples in the German language, employing online surveys as part of a larger research project. Participants were recruited through voluntary organizations and by means of online advertising. In a first wave, we collected Sample 1, and in a second wave, Sample 2 together with Sample 3. For the Sample 3, we recruited professionals who had been interviewed and portrayed in books on the topic of profession and calling (see, for example, Morgenthaler, 2010). They received a personal invitation to participate in the study, and we built a separate data collector for them. The inclusion criterion for the Sample 1 was a minimum age of 50, while Samples 2 and 3 involved a minimum age of 40 years. The participants received no renumeration. Upon completion of the surveys, participants could download brochures with suggestions on how to promote their mental well-being and healthy aging, and create a fulfilling life.

#### Instruments

Table 3 presents all instruments used for this study. Sociodemographic and relevant contextual questions were asked about age, gender, marital status, being a parent, educational level, employment status, financial status, self-rated health, spirituality in daily life, childhood experience, and volunteering.

**TABLE 3:** Instruments used for this study.

Instrument	Authors	Content	Sample item	Number of items	Response format	Use in sample	α in this study
Fulfilled Life item pool	Baumann and Ruch	Cognitive and affective experience of a fulfilled life	“I have led my life in a way that has deeply suited me.”	81 and 12	6-point Likert scale (1 = does not apply at all to 6 = applies completely)	Sample 1	0.87–0.93 (Cognitive facets), 0.96 (Affective Experience)
Fulfilling Life Rating – present*^a^*	Baumann and Ruch (developed for this study)	Fulfilling life (comprising life at the current life stage)	“Compared to your given example, how fulfilling is your life at the current life stage?”	1	11-point Likert scale (0 = not at all fulfilling to 10 = entirely fulfilling)	Sample 1 and 2	–
Fulfilled Life Rating – retrospect*^a^*	Baumann and Ruch (developed for this study)	Fulfilled life (comprising the whole lived life)	“Compared to your given example, how fulfilled is your life lived so far in retrospect?”	1	11-point Likert scale (0 = not at all fulfilled to 10 = entirely fulfilled)	Sample 1 and 2	–
Fulfilled Life Rating – if life ended tomorrow	Baumann and Ruch (developed for this study)	Fulfilled life (comprising the whole lived life)	“If my life were to end tomorrow, I could say with full conviction that my life was …”	1	6-point Likert scale (1 = not fulfilled at all to 6 = completely fulfilled)	Sample 2	–
Orientations to Happiness questionnaire (OTH)*^b^*	Peterson et al. (2005; in the German adaptation by Ruch et al., 2010)	Orientations to well-being: pleasure, engagement, and meaning	“I seek out situations that challenge my skills and abilities.”	15	5-point Likert scale (1 = very much unlike me to 5 = very much like me)	Sample 1	0.72 (Pleasure), 0.68 (Engagement), 0.79 (Meaning)
The short scales for assessing positive relationships and accomplishment	Gander et al., 2017	PERMA dimensions of positive relationships and accomplishment	“A good life means to me that I can share it with others.”	10	5-point Likert scale (1 = very much unlike me to 5 = very much like me	Sample 1	0.74 (Positive Relationships), 0.80 (Accomplishment)
Temporal Satisfaction With Life Scale (TSWLS)	Pavot et al. (1998; in the German adaptation by Trautwein, 2004)	Life satisfaction in the past, present, and future	“My life in the past was ideal for me.”	12	7-point Likert scale (1 = strongly disagree to 7 = strongly agree)	Sample 1 and 2	0.88 (Past Life Satisfaction), 0.89–0.92 (Present Life Satisfaction), 0.90–0.92 (Future Life Satisfaction)
Positive and Negative Affect Schedule (PANAS)	Watson et al. (1988; in the German adaptation by Krohne et al., 1996)	Intensity of positive and negative affect	“Active”	20	5-point Likert scale (1 = very slightly or not at all to 5 = extremely)	Sample 1	0.88 (Positive Affect), 0.87 (Negative Affect)
Lie Scale of the short form of the Eysenck Personality Questionnaire-Revised (EPQ-R)	Eysenck and Eysenck (1991; in the German adaptation by Ruch, 1999)	Social desirability	“Have you ever cheated at a game?”	12	“Yes” or “no” questions	Sample 1	0.72
The Questionnaire for Eudaimonic Well-Being (QEWB)	Waterman et al. (2010; in a German version translated following the standard translation process)	Eudaimonic functioning	“I find a lot of the things I do are personally expressive for me.”	21	5-point Likert scale (0 = strongly disagree to 4 = strongly agree)	Sample 2	0.83
Loyola Generativity Scale (LGS)	McAdams and de St. Aubin (1992; in the German translation reported by Hofer et al., 2008)	Generative concern	“I try to pass along the knowledge I have gained through my experiences.”	20	4-point Likert Scale (0 = not at all to 3 = extremely)	Sample 2	0.86
Ego Integrity Scale (RHEIS)	Ryff and Heincke (1983; in the German translation reported by Busch et al., 2018)	Erikson’s conceptualization of psychological maturity in late adulthood	“In general, I would say I have few regrets about my past life.”	16	6-point Likert Scale (0 = strongly disagree to 5 = strongly agree)	Sample 2	0.90
Serenity subscale of the Positive and Negative Affect Schedule – Extended (PANAS-X)	Watson and Clark (1994; in the German version by Grühn et al., 2010)	Affective state of serenity	“At ease.”	3	5-point Likert Scale (1 = very slightly or not at all to 5 = extremely)	Sample 2	0.88
Warwick-Edinburgh Mental Well-Being Scale (WEMWBS)	Tennant et al. (2007; in the German translation)	Positive mental health during the last 2 weeks	“I’ve been feeling useful.”	14	5-point Likert scale (1 = none of the time to 5 = all of the time)	Sample 2	0.90
Attitude Toward Own Aging subscale of the Philadelphia Geriatric Center Morale Scale	Lawton (1975; in the German translation reported by Beyer et al., 2015)	Self-perceptions of aging	“Things keep getting worse as I get older.”	5	4-point Likert Scale (1 = does not apply at all to 4 = fully applies)	Sample 2	0.72
The Presence subscale of the Brief Calling Scale (BCS)	Dik et al. (2012; in the German translation by Hirschi, 2011)	A sense of a calling in one’s work	“I have a calling to a particular kind of work.”*^c^*	2	5-point Likert scale (1 = not at all true of me to 5 = totally true of me)	Sample 2	0.89*^d^*

*^a^Those ratings were anchored in a short description provided by each participant, in which participants presented their most telling example of a fulfilling and a fulfilled life in a few sentences for each, respectively.*

*^b^This scale was used together with the short scales to assess positive relationships and accomplishment (Gander et al., 2017) to assess all PERMA dimensions of Seligman’s (2011) well-being theory. For this purpose, each OTH scale was reduced by one item.*

*^c^In order to not restrict a calling to paid employment and in consideration that our sample comprises retirees, we added the term activity to both items.*

*^d^Spearman–Brown coefficient.*

## Data Analysis and Results

### Data Analysis

For all statistical analyses, we used IBM SPSS Statistics, version 25.0. Descriptive statistics (mean, standard deviation, minimum, maximum, skewness, kurtosis, corrected item-total correlations) and reliability coefficients (Cronbach’s alpha) were calculated. To investigate the structure of the FLS and determine the number of components aside from the general factor of a fulfilled life, we employed principal component analysis. Since we expected the factors to be correlated, we performed oblique rotation. The number of factors was determined through the use of parallel analysis (Horn, 1965) and the minimum average partial test (MAP; Velicer, 1976), using SPSS syntax written by O’Connor (2000), while also by examining all possible factor solutions between one and nine (according to our nine facet-based scales) through hierarchical factor analysis (Goldberg, 2006). This analysis technique reveals the unfolding of the factors by correlating the factor scores of each level (beginning with the first unrotated principal component [FUPC]) with those of the next level. The procedure facilitates examining whether factors are reorganized or remain stable across different levels. In addition to these extraction criteria, we selected a factor solution that accounted for a substantial proportion of variance and – most importantly – that was plausibly interpretable.

### Results

#### Fulfilled Life Cognitive Experience

##### Preliminary Results

We used Sample 1 to compute total scores for the nine cognitive facets by averaging the assigned items. All nine facets were strongly correlated, which indicated a general factor of a fulfilled life. Cronbach’s alpha ranged between 0.87 and 0.93, and the corrected item-total correlations ranged from 0.28 to 0.84. For economic reasons and as eight facets had between one and three items with a lower corrected-item-total correlation as its correlation with other facets, we selected the best six items per facet (54 items in total) for further analyses and to form a raw version with nine scales. Descriptive statistics, reliability (Cronbach’s alpha) coefficients (Table 4), and zero-order correlations were calculated for these nine raw version scales (Table 5).

**TABLE 4:** Descriptive statistics of fulfilled life facets.

	*M*	*SD*	*S*	*K*	α	citc	FUPC
(A) Realized uniqueness	4.58	0.73	–0.69	0.57	0.82	0.47–0.65	0.81
(B) A life lived fully	4.49	0.80	–0.83	1.12	0.88	0.63–0.76	0.86
(C) The making of a positive difference	4.75	0.72	–0.67	1.57	0.89	0.63–0.77	0.82
(D) Authentic pursuits	4.56	0.79	–0.85	1.29	0.87	0.61–0.73	0.86
(E) A life true to oneself	4.62	0.82	–0.96	1.34	0.89	0.68–0.75	0.87
(F) A contribution reflecting the self	4.55	0.84	–0.50	0.31	0.92	0.58–0.84	0.74
(G) Worthwhile involvements	4.68	0.75	–0.89	1.83	0.90	0.70–0.74	0.90
(H) A life that was worthwhile	4.78	0.79	–1.33	3.00	0.87	0.43–0.82	0.84
(I) A life that mattered to others	4.64	0.81	–0.76	1.58	0.90	0.54–0.81	0.74

*Sample 1 = N_Development_ = 282. Six items per facet.*

*α, Cronbach’s alpha; citc, corrected item total correlation range; FUPC, first unrotated principal component.*

**TABLE 5:** Zero-order correlations fulfilled life facets.

	1	2	3	4	5	6	7	8	9
1 (A) Realized uniqueness	–								
2 (B) A life lived fully	0.71	–							
3 (C) The making of a positive difference	0.62	0.61	–						
4 (D) Authentic pursuits	0.71	0.79	0.59	–					
5 (E) A life true to oneself	0.67	0.78	0.60	0.83	–				
6 (F) A contribution reflecting the self	0.49	0.50	0.74	0.50	0.55	–			
7 (G) Worthwhile involvements	0.67	0.76	0.64	0.76	0.79	0.60	–		
8 (H) A life that was worthwhile	0.65	0.69	0.60	0.68	0.68	0.50	0.79	–	
9 (I) A life that mattered to others	0.52	0.51	0.74	0.43	0.49	0.67	0.62	0.61	–

*Sample 1 = N_Development_ = 282. Six items per facet. All correlations p < 0.001.*

Table 4 reveals that all scales were negatively skewed, suggesting that the respondents favorably rated their lives as fulfilling. All scales had good internal consistency, with alpha coefficients ranging from 0.82 to 0.92.

Table 5 demonstrates that all raw version scales were positively intercorrelated, indicating a g-factor of cognitive fulfillment. The facets of the cells A, B, D, and E were highly intercorrelated, representing the sources self and life in relation to the criteria completeness/wholeness and congruence. The high correlation between cells D and E, which had different sources but the same criterion, might suggest that the criterion had greater importance than the sources. The same applies to the correlation between G and H. In addition, G and H were also strongly related to the cells B, D, and E, sharing the same sources. Higher correlations could also be found for the cells C, F, and I, which represented the facets of the column impact/legacy and were related due to their common source. Thus, the pattern of the 3 × 3 matrix seemed to exist within the data and reflected the characteristics of the model. Nevertheless, further examination was required to find a structure.

We submitted the pool of 54 items to principal component analysis to investigate the underlying factor structure. The Kaiser–Meyer–Olkin measure verified the sampling adequacy for the analysis, KMO = 0.96, which exceeded the minimum criteria of 0.5 (Hutcheson and Sofroniou, 1999), and Bartlett’s test of sphericity suggested that the data were suitable for factor analysis, χ^2^(1431) = 11,990.29, *p* < 0.001. Eight factors exceeded unity (the first ten eigenvalues were 23.69, 4.24, 2.19, 1.62, 1.51, 1.20, 1.11, 1.01, 0.93, 0.85). The parallel analysis yielded three components with eigenvalues exceeding the randomly generated values (95% CI, 1000 random correlation matrices; see O’Connor, 2000), and MAP suggested a seven-factor solution. In summary, the different tests did not suggest the extraction of the same number of factors.

##### Hierarchical Factor Analysis

**FIGURE 1:**
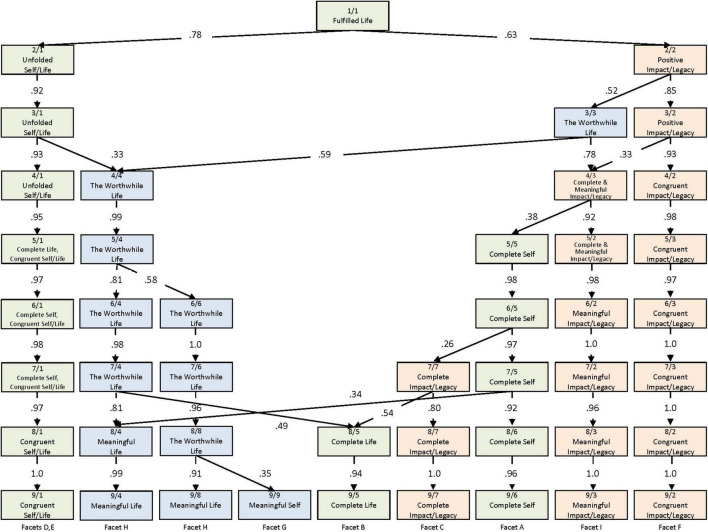
Hierarchical factor analysis of the fulfilled life item set of the cognitive component. Hierarchical representation of the emergence of the first nine varimax-rotated principal components derived from the 54 cognitive items of the FLS. Correlations between the factors of adjacent levels are represented using arrows (only correlations > | 0.30| are shown). The color of the squares indicates the assignment of the factors from level 3 onward.

In seeking to arrive at the best factor-solution at the item level, we examined all solutions for between one and nine factors by employing hierarchical factor analysis (Goldberg, 2006). Using Sample 1, we performed principal component analyses with varimax rotation. Figure 1 illustrates the resulting hierarchical structure.

In the overview, the factors are designated by their size (denoted by the number to the right of the factor; e.g., 3/1 is the largest and 3/3 the smallest factor at that level), which is the amount of explained variance by that factor. The figure further shows the correlations between the factor scores and those at adjacent levels. In addition, we correlated the factors at each level with the nine facets (average scores) to examine the correspondence between the facets and the derived factors.

All items loaded on the first unrotated principal component, which represented the general factor of a Fulfilled Life. At the second level, the first unrotated principal component was divided into one factor (2/1), labeled Unfolded Self and Life, and a smaller second factor (2/2), deemed Positive Impact and Legacy. The first factor was strongly loaded by items of the six facets that referred to the columns Self and Life of the model, and the second factor was mainly loaded by the three Impact/Legacy cells and partly by G and H. At the third level, the items of the facets G and H that had previously loaded on both factors now formed a new factor: The Worthwhile Life. This three-factor solution offered a rearrangement of the nine facets. The factor Unfolded Self and Life (3/1) covered mainly the facet of Self in terms of the criterion congruence and also the two facets Life regarding the criteria of completeness and congruence. The factor Positive Impact and Legacy (3/2) consisted of high loadings from items of all three facets concerning Impact/Legacy. Whereas, The Worthwhile Life (3/3) contained high loadings from items of the facets in terms of the criterion meaningfulness. The evolution of the factors revealed, especially with respect to the sources Self and Life, a separation between the two criteria completeness and congruence and the criterion of meaningfulness.

In the next six steps, the factors divided further. It should be mentioned before continuing the discussion that at level nine, only six of the nine factors represented a facet from the model; one factor combined two facets, and two factors represented the same single facet. Thus, the evident redundancy among the facets suggested that a lower number might be more suitable. More specifically, the factor Unfolded Self and Life divided into two factors after level four, building a new factor (5/5) together from the division of the factor (4/3). At the eighth level, three factors emerged, and these remained the same also at the ninth level. The three factors (9/1, 9/5, 9/6) had high item loadings from the facets Self and Life relating to the criteria completeness and congruence. However, those factors did not correspond completely to the facets since two facets were combined in one factor (9/1). Nevertheless, these results support the notion of sources and criteria in the model, as for the factor Congruent Self/Life (9/1), the criteria seem to have a greater effect on the factor than the sources.

Positive Impact and Legacy divided into two factors at the fourth level, into three at the seventh level, and remained unchanged all the way through the ninth level. These three resulting factors represent the columns impact and legacy exactly, comprising the three facets representing the source and one of the three criteria.

At the fourth level, The Worthwhile Life was mainly represented by items from the Meaningful Self and Life facets. However, it split up into two factors at the sixth level and into three at the ninth level, whereby two factors represented the Meaningful Life and one factor the Meaningful Self, as already mentioned.

In summary, the pattern of the nine facets became evident and presented a justification of the 3 × 3 matrix, but not all of them could be separated. Though a solution with a higher number of factors could account for more variance, a three-factor solution appeared to be the most interpretable and relevant for research and application. Selecting a four-factor solution in which Positive Impact and Legacy was divided into two factors might not have led to additional value, as the one factor already represented a well-rounded concept. The advantage of having conducted a hierarchical factor analysis compared to solely performing EFAs lay in the ability to separate the two modes of the model (sources and criteria) and gain a more comprehensive understanding of the structural representation of the nine cognitive facets.

##### Exploratory Factor Analysis

Based on the decision to retain three factors, we performed a principal component analysis with oblique (oblimin direct with delta = 0) rotation on Sample 1. The three factors explained 55.78% of the variance. For economic reasons and to create provisional scales with an equal number of items, we selected the best eight items per factor by considering factor loadings, construct representation, and avoidance of content overlap among the selected items. We subjected the reduced item set (24 items) to new principal component analysis. Three eigenvalues exceeded unity (the first five eigenvalues were 10.79, 2.32, 1.57, 0.99, 0.85). Again, oblique (oblimin direct with delta = 0) rotation was performed. The three factors explained 61.19% of the variance, and factor loadings ranged from 0.46 to 0.85. Table 6 exhibits the new pattern matrix and the extracted communalities. Unfolded Self and Life correlated with Positive Impact/Legacy at *r* = 0.35 and with The Worthwhile Life at *r* = 0.45. Positive Impact/Legacy correlated with the Worthwhile Life at *r* = 0.49.

**TABLE 6:** Pattern matrix of the three-factor principal component analyses with oblimin rotation.

Items	Items in English	Sample 1				Sample 2			
		F1	F2	F3	*h* ^2^	F1	F2	F3	*h* ^2^
Factor 1: Unfolded Self and Life									
(2) Ich konnte meine Einzigartigkeit zeigen. (A)	I could show my uniqueness.	**0.60**	0.22	0.06	0.46	**0.74**	0.25	0.21	0.61
(5) Ich konnte mein wahres Können im Leben zeigen. (A)	I was able to show my true ability in life.	**0.61**	0.24	0.06	0.58	**0.77**	0.11	0.04	0.65
(8) Ich habe meine Chancen im Leben genutzt. (B)	I took advantage of my opportunities in life.	**0.46**	0.13	0.30	0.51	**0.67**	0.01	0.14	0.59
(12) Ich konnte eigene Träume verwirklichen. (B)	I could realize my own dreams.	**0.85**	0.02	0.03	0.71	**0.69**	0.10	0.25	0.66
(20) Ich habe den Mut gehabt, so zu sein, wie ich wirklich bin. (D)	I have had the courage to be as I really am.	**0.65**	0.11	0.19	0.51	**0.76**	0.01	0.04	0.60
(21) Ich habe meinen Leidenschaften nachgehen können. (D)	I have been able to pursue my passions.	**0.81**	0.02	0.02	0.66	**0.83**	0.06	0.04	0.69
(26) Ich konnte im Leben das tun, wofür ich am besten geeignet war. (E)	I could do in life that which I was best suited for.	**0.66**	0.14	0.16	0.66	**0.82**	0.06	0.00	0.72
(29) Ich habe mein Leben so geführt, wie es mir zutiefst entsprochen hat. (E)	I have led my life in a way that has deeply suited me.	**0.65**	0.01	0.32	0.70	**0.74**	0.10	0.21	0.70
Factor 2: Positive Impact/Legacy									
(13) Ich habe Möglichkeiten genutzt, um zum Wohlergehen anderer beizutragen. (C)	I have used opportunities to contribute to others’ well-being.	0.05	**0.82**	0.09	0.64	0.02	**0.82**	0.01	0.69
(16) Ich konnte mit meinem Leben eine positive Spur bei Menschen in meinem Umfeld hinterlassen. (C)	I was able to leave a positive mark with my life on people in my environment.	0.11	**0.63**	0.17	0.62	0.17	**0.62**	0.11	0.60
(18) Ich konnte einen positiven Beitrag zum Wohle anderer Menschen leisten. (C)	I could make a positive contribution to other people’s welfare.	0.09	**0.76**	0.01	0.65	0.08	**0.79**	0.04	0.72
(34) Es war mir ein Anliegen, etwas zum Gelingen unserer Gesellschaft beizutragen. (F)	It was important to me to contribute something to the success of our society.	0.11	**0.74**	0.09	0.55	0.00	**0.84**	0.15	0.63
(36) Ich habe meine Fähigkeiten genutzt, um einen Beitrag für das Allgemeinwohl zu leisten. (F)	I have used my abilities to make a contribution to the common good.	0.20	**0.80**	0.12	0.70	0.11	**0.84**	0.06	0.75
(51) Ich habe andere Menschen in ihrer Entwicklung massgeblich unterstützt. (I)	I have significantly supported other people in their development.	0.17	**0.63**	0.35	0.63	0.07	**0.80**	0.11	0.67
(52) Ich konnte zum Gelingen des Lebens anderer Menschen beitragen. (I)	I could contribute to the success of other people’s lives.	0.27	**0.64**	0.37	0.64	0.05	**0.81**	0.12	0.71
(54) Ich habe für einen Zweck gelebt, der über mein Leben hinausgeht. (I)	I have lived for a purpose that goes beyond my life.	0.00	**0.51**	0.27	0.47	0.05	**0.60**	0.26	0.53
Factor 3: The Worthwhile Life									
(39) Die Anstrengungen im Leben haben sich gelohnt. (G)	The efforts in life have been worthwhile.	0.13	0.08	**0.67**	0.61	0.09	0.04	**0.75**	0.67
(40) Ich habe die Gewissheit, dass ich für die richtigen Dinge gelebt habe. (G)	I have the certainty that I have lived for the right things.	0.23	0.08	**0.64**	0.66	0.23	0.16	**0.58**	0.68
(43) Ich habe etwas Wertvolles mit meinem Leben gemacht. (H)	I have done something valuable with my life.	0.12	0.29	**0.53**	0.61	0.17	0.33	**0.54**	0.73
(44) Ich kann auf ein gut gelebtes Leben zurückblicken. (H)	I can look back on a life well lived.	0.41	0.12	**0.62**	0.68	0.28	0.04	**0.69**	0.74
(45) Auch für die schwierigen Zeiten im Leben habe ich Bedeutung und Sinn erkennen können. (H)	Even in the difficult times in life, I have been able to recognize meaning and purpose.	0.10	0.09	**0.74**	0.55	0.10	0.01	**0.76**	0.51
(46) Mein Leben hat sich gelohnt. (H)	My life has been worthwhile.	0.10	0.01	**0.83**	0.79	0.14	0.01	**0.81**	0.80
(47) Ich habe mein Leben als sinnvoll erfahren. (H)	I have experienced my life as meaningful.	0.18	0.00	**0.81**	0.82	0.20	0.05	**0.73**	0.78
(48) Ich habe erkannt, worauf es im Leben wirklich ankommt. (H)	I have realized what really matters in life.	0.01	0.00	**0.55**	0.30	0.04	0.20	**0.60**	0.47

*Sample 1 = N_Development_ = 282. Sample 2 = N_Replication_ = 406.*

*Bold loadings indicate the factor on which the item was retained. The letter in brackets indicates the original facet of the model (Find the meaning of the letters in Table 1). English items were translated from the original German items employing a translation/back translation procedure. The translation has not been validated.*

#### Fulfilled Life Affective Experience

##### Exploratory Factor Analysis

In this process, we first submitted the 12 items for affective fulfillment to principal component analysis using Sample 1. The negatively worded items were recoded beforehand. To verify the sampling adequacy for the analysis, we used the Kaiser–Meyer–Olkin measure, KMO = 0.95, which was highly acceptable. Bartlett’s test suggested that the data were suitable for factor analysis, χ^2^(66) = 3,278.31, *p* < 0.001. Two eigenvalues exceeded unity; the first four eigenvalues were 8.27, 1.04, 0.51, 0.47. Parallel analysis indicated a one-factor solution and the MAP test a two-factor solution. For economic reasons and due to the high internal consistency of the Fulfilled Life Affective Experience scale with Cronbach’s alpha of 0.96, we selected the best eight items (five positively worded items and three recoded items) to create a one-dimensional scale. Due to its highly positively skewed distribution, the item “I feel empty” was slightly changed to “I feel rather empty.” To evaluate the suggested one-factor structure of the eight items, we conducted a principal component analysis. The PCA revealed a one-factor structure (the first three eigenvalues were 5.46, 0.80, 0.49). The first factor explained 68.30% of the variance. Parallel analysis and MAP equally suggested a one-factor solution. The factor loadings ranged from 0.67 to 0.90 (see Table 7).

**TABLE 7:** Factor loadings of fulfilled life affective items.

Items	Items in English	Sample 1	Sample 2
		
		Factor loadings	Factor loadings
(1) verspüre ich eine tiefe innere Zufriedenheit.	I feel deep inner contentment.	0.87	0.85
(2) fühle ich mich im Einklang mit mir und dem gelebten Leben.	I feel in harmony with myself and the lived life.	0.89	0.87
(3) habe ich einen inneren Frieden.	I have inner peace.	0.88	0.87
(5) empfinde ich grosse Dankbarkeit.	I feel great gratitude.	0.71	0.81
(6) fühle ich mich erfüllt.	I feel fulfilled.	0.90	0.89
(7) empfinde ich tiefe Reue. (R)	I feel deep regret. (R)	0.67	0.55
(11) fühle ich mich enttäuscht. (R)	I feel disappointed. (R)	0.82	0.81
(12) fühle ich mich eher leer. (R)	I feel rather empty. (R)	0.84	0.82

*Sample 1 = N_Development_ = 282. Sample 2 = N_Replication_ = 406. Reverse-scored items are denoted with (R).*

##### Descriptive Statistics

Total scores for all scales were computed by averaging the assigned items. Descriptive statistics, internal consistencies of the FLS scales, and their correlations with age and gender in Sample 1 are presented in Table 8. The scales were negatively skewed, suggesting that the respondents appraised their lives, in general, and the worthwhileness of their lives, in particular, as fulfilling. Skewness ranged from –0.63 to –1.38, and kurtosis ranged from 0.90 to 3.37. The internal consistencies of the scales were high (α = 0.89–0.94). Corrected item-total correlations (not included in Table 8) varied from 0.44 to 0.85 for the cognitive items and from 0.59 to 0.85 for the affective items. Results revealed only for affective fulfillment and age a small positive correlation (*r* = 0.15, *p* < 0.05). No significant association emerged between the scales and gender. Meanwhile, the FLSs were strongly intercorrelated (see Table 9). The Fulfilled Life Scale (FLS) and scoring information are available in the Supplementary Material of this article.

**TABLE 8:** Descriptive statistics, internal consistencies of the fulfilled life scales and correlations of the fulfilled life scales with age and gender.

Scale	Descriptive statistics and internal consistencies								Correlations	
	*N items*	*Min*	*Max*	*M*	*SD*	*S*	*K*	α	Age	Gender
Sample 1										
USL	8	1.38	6.00	4.34	0.80	–0.74	0.90	0.90	0.06	–0.11
PIL	8	1.38	6.00	4.64	0.73	–0.63	1.01	0.89	0.10	0.01
TWL	8	1.00	6.00	4.76	0.78	–1.38	3.37	0.90	0.11	0.04
FLCE	24	1.75	6.00	4.58	0.67	–0.87	1.38	0.94	0.10	–0.03
FLAE	8	1.38	6.00	4.77	0.91	–1.25	1.58	0.93	0.15*	0.01
Sample 2										
USL	8	1.25	6.00	4.28	0.90	–0.64	0.38	0.92	0.10	0.01
PIL	8	1.00	6.00	4.59	0.85	–0.87	1.28	0.91	0.15**	0.09
TWL	8	1.13	6.00	4.71	0.84	–1.08	1.66	0.92	0.16**	0.06
FLCE	24	1.75	5.92	4.53	0.74	–0.75	0.75	0.95	0.15**	0.06
FLAE	8	1.00	6.00	4.73	0.94	–1.15	1.16	0.92	0.20***	0.11*

*Sample 1 = N_Development_ = 280–282. Sample 2 = N_Replication_ = 405–406. Scale range: 1–6.*

*S, Skewness; K, Kurtosis; α, Cronbach’s alpha. Male = 1, female = 2. FLCE, Fulfilled Life Cognitive Experience; USL, Unfolded Self and Life; PIL, Positive Impact and Legacy; TWL, The Worthwhile Life; FLAE, Fulfilled Life Affective Experience.*

*p < 0.05; **p < 0.01; ***p < 0.001.

**TABLE 9:** Pearson correlations of the fulfilled life scales.

	1	2	3	4	5
Sample 1					
(1) USL	–				
(2) PIL	0.54	–			
(3) TWL	0.70	0.65	–		
(4) FLCE	0.87	0.83	0.90	–	
(5) FLAE	0.69	0.48	0.80	0.76	–
Sample 2					
(1) USL	–				
(2) PIL	0.52	–			
(3) TWL	0.72	0.60	–		
(4) FLCE	0.87	0.82	0.90	–	
(5) FLAE	0.71	0.46	0.85	0.78	–

*Sample 1 = N_Development_ = 282. Sample 2 = N_Replication_ = 406.*

*USL, Unfolded Self and Life; PIL, Positive Impact and Legacy; TWL, The Worthwhile Life; FLCE, Fulfilled Life Cognitive Experience; FLAE, Fulfilled Life Affective Experience.*

*All correlations p < 0.001.*

### Phase 3: Structural Validity II

#### Data Analysis

All statistical analyses used IBM SPSS Statistics (Version 25), except for confirmatory factor analyses, which were conducted with the lavaan package for R. The analyses were based on Sample 2. The following goodness-of-fit indices were applied to evaluate the CFA model: values ≥ 0.90 in the comparative fit index (CFI) and Tucker–Lewis index (TLI; Hu and Bentler, 1999), value ≤ 0.08 in the root mean square error of approximation (RMSEA; Browne and Cudeck, 1992), and value ≤ 0.08 in the standardized root mean residual (SRMR; Hu and Bentler, 1999).

#### Results

##### Fulfilled Life Cognitive Experience

The cognitive items of the final version of the FLS were first subjected to principal component analysis. The KMO index was 0.96, and the Bartlett’s test of sphericity suggested that the data were adequate for factor analysis, χ^2^(276) = 7,147.21, *p* < 0.001. Three factors exceeded unity (the first five eigenvalues were 11.60, 2.76, 1.51, 0.77, 0.67). We extracted three factors and performed an oblique (oblimin direct with delta = 0) rotation. The three factors explained 66.13% of the variance. The intercorrelations of the factors were: *r* = 0.44 for Unfolding of the Self and Life and Positive Impact and Legacy, *r* = 0.57 for Unfolding the Self and Life and The Worthwhile Life, and *r* = 0.42 for Positive Impact and Legacy and The Worthwhile Life. The new pattern matrix, displayed in Table 6, shows that all items had their highest loading on the intended factor. The factor loadings ranged from 0.54 to 0.84, with cross-loading differences > 0.20. Tucker’s Phi coefficients indicated that the extracted factors were similar across the two samples: Unfolded Self/Life: φ = 0.97, Positive Impact/Legacy: φ = 0.98, The Worthwhile Life: φ = 0.96. Lastly, the three-factor model was evaluated by confirmatory factor analysis with robust estimator: model fit: χ^2^(249) = 636.92, *p* < 0.001; all other fit indices indicated an acceptable fit to the data (CFI = 0.931, TLI = 0.924, RMSEA = 0.069, SRMR = 0.060). The robustness of the three-factor structure determined from the EFA of Sample 1 was supported. Information on item statistics is provided in Supplementary Table A1.

##### Fulfilled Life Affective Experience

We first subjected the items of the final version to principal component analysis. The KMO index was 0.94, and Bartlett’s test of sphericity indicated that the data were adequate for factor analysis, χ^2^(28) = 2,319.21, *p* < 0.001. One factor exceeded unity (the first three eigenvalues were 5.31, 0.83, 0.49). The one-factor explained 66.36% of the variance. The factor loadings ranged from 0.55 to 0.89 (see Table 7). Tucker’s Phi coefficient indicated that the factor was similar across the two samples (φ = 1.00). To determine the model quality, we conducted confirmatory factor analysis with a robust estimator. Model fit was χ^2^(20) = 73.46, *p* < 0.001; all other fit indices indicated an adequate fit to the data (CFI = 0.969, TLI = 0.957, RMSEA = 0.093, SRMR = 0.040). Information on item statistics is presented in the Supplementary Table A.

##### Descriptive Statistics

Table 8 presents descriptive statistics, internal consistencies of the FLS scales, and their correlations with age and gender. The table shows that the means were above the scale’s midpoint and that the standard deviations were higher in the replications sample. Skewness ranged from –0.64 to –1.15, and kurtosis ranged from 0.38 to 1.66. The internal consistencies of the scales were high (α = 0.91–0.95). Small positive correlations between all scales (except Unfolded Self and Life) and age were found. Women reported slightly higher affective fulfillment than men. Overall, the scales were strongly intercorrelated (see Table 9).

### Phase 4: External Validity

For the evaluation of construct and criterion validitiy, analyses were conducted on Sample 1 and 2 separately. We partly used different instruments in the samples. Results of convergent, discriminant, and concurrent validity can be found in Table 10, while results for incremental validity are displayed in Table 11.

**TABLE 10:** Descriptive statistics, intercorrelations, convergent, discriminant, and concurrent validity of the FLS subscales.

Measures	Sample 1							Sample 2						
	*M*	*SD*	USL	PIL	TWL	FLCE	FLAE	*M*	*SD*	USL	PIL	TWL	FLCE	FLAE
*Convergent validity*														
Fulfilling life rating – present	7.41	1.94	0.52***	0.39***	0.61***	0.59***	0.71***	−	−	−	−	−	−	−
Fulfilled life rating – retrospect	7.59	1.77	0.66***	0.46***	0.64***	0.68***	0.70***	7.64	1.78	0.64***	0.36***	0.70***	0.66***	0.74***
Fulfilled life rating – if life ended tomorrow	−	−	−	−	−	−	−	4.77	0.91	0.64***	0.38***	0.76***	0.69***	0.80***
Life satisfaction past	4.68	1.27	0.53***	0.24***	0.47***	0.48***	0.49***	4.55	1.36	0.52***	0.12*	0.39***	0.40***	0.46***
PERMA														
Pleasure	3.06	0.70	0.31***	0.20**	0.28***	0.31***	0.22***	−	−	−	−	−	−	−
Engagement	3.24	0.68	0.43***	0.39***	0.42***	0.48***	0.34***	−	−	−	−	−	−	−
Positive relationships	3.37	0.72	0.17**	0.28***	0.18**	0.24***	0.09	−	−	−	−	−	−	−
Meaning	3.10	0.83	0.33***	0.58***	0.51***	0.54***	0.36***	−	−	−	−	−	−	−
Accomplishment	3.39	0.74	0.42***	0.38***	0.41***	0.47***	0.26***	−	−	−	−	−	−	−
EWB	−	−	−	−	−	−	−	62.08	9.07	0.58***	0.51***	0.68***	0.69***	0.62***
Generativity	−	−	−	−	−	−	−	36.32	8.79	0.51***	0.76***	0.56***	0.70***	0.45***
Ego integrity	−	−	−	−	−	−	−	56.68	12.90	0.67***	0.36***	0.71***	0.67***	0.77***
Positive affect	34.86	6.18	0.54***	0.50***	0.57***	0.62***	0.57***	−	−	−	−	−	−	−
Serenity	−	−	−	−	−	−	−	10.30	2.38	0.47***	0.26***	0.55***	0.50***	0.59***
*Discriminant validity*														
Social desirability	2.26	2.07	0.06	0.01	0.02	0.03	0.02	−	−	−	−	−	−	−
NA	15.95	5.59	−0.30***	−0.14*	−0.31***	−0.28***	−0.48***	−	−	−	−	−	−	−
*Concurrent validity*														
Life satisfaction present	5.50	1.16	0.51***	0.36***	0.55***	0.55***	0.65***	5.37	1.27	0.52***	0.28***	0.66***	0.57***	0.68***
Life satisfaction future	5.38	1.00	0.28***	0.28***	0.37***	0.36***	0.34***	5.46	1.11	0.36***	0.29***	0.58***	0.47***	0.56***
Mental well-being	−	−	−	−	−	−	−	54.69	7.18	0.58***	0.39***	0.67***	0.63***	0.67***
Self-perceptions of aging	−	−	−	−	−	−	−	15.47	2.75	0.37***	0.30***	0.52***	0.46***	0.54***
Calling	−	−	−	−	−	−	−	7.42	2.18	0.45***	0.46***	0.45***	0.53***	0.39***

*Sample 1 = N_Development_ = 282, Sample 2 = N_Replication_ = 406*

*USL, Unfolded Self and Life; PIL, Positive Impact and Legacy; TWL, The Worthwhile Life; FLCE, Fulfilled Life Cognitive Experience; FLAE, Fulfilled Life Affective Experience; EWB, Eudaimonic Well-being; NA, Negative Affect.*

*p < 0.05; **p < 0.01; ***p < 0.001.

**TABLE 11:** Hierarchical regression predicting the fulfilled life rating – retrospect and mental well-being.

Variable	Predictor	Step 1				Step 2				Step 3				Step 4			
		*B*	95% CI	*SE B*	β	*B*	95% CI	*SE B*	β	*B*	95% CI	*SE B*	β	*B*	95% CI	*SE B*	β
Fulfilled life rating – retrospect	Constant	6.01	[4.52, 7.47]	0.75		2.10	[0.35, 4.18]	0.92		1.04	[–1.18, 3.53]	1.12		–1.92	[–3.87,0.18]	0.91	
	Age	0.04	[0.01,0.06]	0.01	0.18**	0.02	[0.00,0.03]	0.01	0.08	0.02	[0.01,0.04]	0.01	0.12*	0.02	[0.00,0.03]	0.01	0.09*
	Gender*^a^*	–0.36	[–0.81,0.12]	0.24	–0.08	–0.31	[–0.63,0.02]	0.17	–0.07	–0.29	[–0.61,0.04]	0.18	–0.07	–0.30	[–0.62,0.04]	0.16	–0.07
	Life satisfaction present					0.44	[0.17,0.69]	0.12	0.29***	0.43	[0.20,0.65]	0.11	0.28***	0.08	[–0.13,0.31]	0.12	0.05
	Positive affect					0.09	[0.06,0.13]	0.02	0.33***	0.09	[0.05,0.13]	0.02	0.33***	0.04	[0.01,0.08]	0.02	0.16*
	Negative affect					–0.04	[–0.08,–0.01]	0.02	–0.12*	–0.05	[–0.08,–0.02]	0.02	–0.15**	0.00	[–0.03,0.03]	0.02	0.01
	Pleasure									–0.01	[–0.30,0.29]	0.14	–0.01	0.00	[–0.23,0.24]	0.12	0.00
	Engagement									–0.32	[–0.67,0.03]	0.18	–0.12	–0.30	[–0.62,0.00]	0.16	–0.12*
	Positive relationships									0.32	[0.04,0.60]	0.15	0.13*	0.26	[0.03,0.49]	0.13	0.11*
	Meaning									0.00	[–0.28,0.28]	0.13	0.00	–0.32	[–0.54, –0.10]	0.12	–0.15**
	Accomplishment									0.22	[–0.13,0.56]	0.19	0.09	0.04	[–0.25,0.29]	0.15	0.02
	FLCE													0.98	[0.38,1.54]	0.28	0.37***
	FLAE													0.72	[0.34,1.12]	0.23	0.37***
	*R* ^2^				0.04				0.39				0.41				0.59
	Δ*R*								0.35***				0.02				0.18***
Mental well-being	Constant	49.60	[44.62, 54.41]	2.51		26.18	[20.73,31.49]	2.69		19.29	[13.93,24.32]	2.64		20.55	[15.63,25.73]	2.64	
	Age	0.08	[0.02,0.16]	0.04	0.12*	0.06	[0.01,0.12]	0.03	0.09*	0.03	[–0.02,0.08]	0.03	0.04	0.01	[–0.04,0.06]	0.02	0.02
	Gender*^a^*	0.15	[–1.51,1.78]	0.87	0.01	–0.28	[–1.43,0.85]	0.63	–0.02	–0.45	[–1.57,0.62]	0.58	–0.03	–0.77	[–1.87,0.27]	0.57	–0.04
	Life satisfaction past					0.59	[0.12,1.06]	0.24	0.11**	0.52	[0.07,0.98]	0.22	0.10**	0.09	[–0.38,0.56]	0.25	0.02
	Life satisfaction present					2.27	[1.69,2.80]	0.30	0.40***	1.89	[1.32,2.42]	0.30	0.34***	1.34	[0.70,1.94]	0.34	0.24***
	Life satisfaction future					1.93	[1.19,2.74]	0.36	0.30***	1.32	[0.63,2.05]	0.36	0.20***	1.00	[0.31,1.76]	0.35	0.16**
	Eudaimonic well-being									0.24	[0.18,0.31]	0.03	0.31***	0.13	[0.05,0.20]	0.04	0.16**
	FLCE													1.62	[0.40,2.84]	0.58	0.17**
	FLAE													1.37	[0.37,2.50]	0.54	0.18**
	*R* ^2^				0.02				0.46				0.53				0.56
	Δ*R*								0.44***				0.07***				0.03***

*Hierarchical regression predicting the fulfilled life rating – retrospect using Sample 1 = N_Development_ = 280, hierarchical regression predicting mental well-being using Sample 2 = N_Replication_ = 404. FLCE, Fulfilled Life Cognitive Experience; FLAE, Fulfilled Life Affective Experience.*

*^a^Male = 1, female = 2. CI = confidence interval. Confidence interval and standard errors based on 1000 bootstrap samples.*

*p < 0.05; **p < 0.01; ***p < 0.001.

#### Convergent and Discriminant Validity

The three global fulfilled life ratings were positively associated with all FLS dimensions. Numerically higher correlations were found for the two ratings from a retrospective view compared to the one assessing the present. While affective fulfillment yielded the strongest relationships with all three ratings, Positive Impact and Legacy had the lowest correlation.

Retrospective life satisfaction positively correlated with all FLS dimensions and yielded the lowest correlation for the Positive Impact and Legacy. The correlation pattern of the PERMA dimensions and the FLSs showed the strongest relationships to engagement, meaning, and accomplishment. Eudaimonic functioning was positively related to all fulfilled life dimensions. Generativity yielded the largest correlation for Positive Impact and Legacy, while ego integrity showed the largest correlation with The Worthwhile Life and affective fulfillment. Serenity was positively related to all FLS dimensions, with the highest correlation for affective fulfillment, as expected. Large positive correlations between positive affect and the fulfilled life dimensions were found. The FLS subscales had no correlations with social desirability and small to moderate negative correlations with negative affect.

#### Concurrent and Incremental Validity

All fulfillment dimensions were related to current and prospective life satisfaction, mental well-being, self-perceptions of aging, and calling. In most cases, we found the highest correlations for The Worthwhile Life and affective fulfillment.

The first hierarchical regression analysis based on Sample 1 revealed that, after controlling for age and gender, cognitive and affective fulfillment could significantly predict variance in a global fulfilled life rating above and beyond subjective well-being and PERMA. When trying to predict the global fulfilled life rating – in retrospect, we entered age and gender in Step 1, which explained 4% of the variance. Next, we added life satisfaction and positive and negative affect (i.e., the variables defining subjective well-being) in Step 2, which increased the prediction by 35%. In Step 3, the PERMA dimensions caused a further increment of 2%. Entering cognitive and affective fulfillment in Step 4 yielded an additional 18% of variance. Cognitive and affective fulfillment turned out to be the strongest predictors (β = 0.37, *p* < 0.001, for both). This outcome means that both subjective and eudemonic (PERMA) well-being do not fully account for global subjective fulfillment, but measured fulfillment is also needed.

To examine whether measured fulfillment might have incremental validity over and above established predictors when predicting important life outcomes, we performed an additional hierarchical regression analysis on Sample 2. The results demonstrated that affective and cognitive fulfillment incrementally predicted mental well-being beyond the temporal life satisfaction scales and eudaimonic well-being and after controlling for age and gender. In Step 1, age and gender were entered first, explaining 2% of the variance; in Step 2, the temporal life satisfaction scales increased the prediction by 44%. In Step 3, the eudaimonic well-being caused a further increment by 7%, and in Step 4, cognitive and affective fulfillment contributed an additional 3% of the variance.

#### Known-Groups Validity

We performed *t*-tests to compare the general sample (Sample 2) with a sample of calling exemplars (Sample 3) regarding the level of the presence of a calling and the experience of a fulfilled life. As expected, the exemplars were significantly more likely to report the presence of a calling (*M* = 8.46, *SD* = 1.62) than persons from the general sample (*M* = 7.42, *SD* = 2.18). This difference, –1.04, BCa 95% CI [–1.527, –0.378], was significant *t*(52.244) = 3.71, *p* = 0.001 (the *t*-test is reported with equal variance not assumed) and represented a medium-sized effect, Hedges’ *g* = 0.49. The groups also differed regarding the level of a fulfilled life on all dimensions (see Table 12), with the calling exemplar reporting significantly greater fulfillment than the general sample. The Unfolded Self and Life yielded the greatest difference, representing a large effect.

**TABLE 12:** Independent samples *t*-test comparing a general and calling exemplar group.

Scale	General		Calling exemplar		*t*	*df*	*p*	BCa 95% CI	Hedges’ *g*
	*M*	*SD*	*M*	*SD*					
USL	4.28	0.90	5.01	0.54	–7.434	60.343	<0.001	[–0.909, –0.536]	0.83
PIL	4.60	0.85	4.91	0.59	–3.059	54.522	0.003	[–0.521, –0.096]	0.38
TWL	4.71	0.84	5.09	0.52	–4.037	58.886	<0.001	[–0.561, –0.188]	0.46
FLCE	4.53	0.74	5.00	0.43	–6.056	62.556	<0.001	[–0.630, –0.312]	0.65
FLAE	4.73	0.94	5.10	0.61	–3.383	56.884	0.001	[–0.584, –0.154]	0.40

*Sample 2 = N_Replication_ = 406 (general sample) and Sample 3 = N = 36 (calling exemplar sample).*

*USL, Unfolded Self and Life; PIL, Positive Impact and Legacy; TWL, The Worthwhile Life; FLCE, Fulfilled Life Cognitive Experience; FLAE, Fulfilled Life Affective Experience. BCa 95% CI for mean difference. t-tests are reported with equal variance not assumed.*

#### Relationships With Sociodemographic and Contextual Characteristics

We examined the relationships between the fulfilled life dimensions and the global rating, along with various sociodemographic and contextual characteristics in the merged sample (see Table 13). Generally, the partial correlations (controlled for age and gender) were meaningful, in the right direction, and small in size. Table 13 displays age effects for the global rating and all fulfilled life dimensions. No significant relationship was found for gender. Better educated individuals reported higher levels in the global rating and all dimensions with the numerically highest coefficient for the Unfolded Self and Life and the lowest coefficient for The Worthwhile Life. In contrast, whether individuals were employed or retired was not associated with fulfillment. Persons in a financially better position reported higher levels of fulfillment in all dimensions, except for Positive Impact/Legacy. Volunteers, in comparison to non-volunteers, had higher scores on the dimensions of Positive Impact and Legacy and cognitive fulfillment. Being married went along with higher levels of fulfillment in the global rating and all dimensions, except in the Unfolded Self and Life and Positive Impact and Legacy. Furthermore, we found significant correlations for parenthood and the global rating, Positive Impact and Legacy, The Worthwhile Life, and cognitive fulfillment. Conversely, the number of children was solely associated with Positive Impact and Legacy. There was a slight positive correlation between being spiritual in daily life and fulfillment in all dimensions, except for the global rating and Unfolded Self and Life. Individuals with better self-evaluated health reported higher levels of fulfillment in the global rating and all dimensions. Finally, a good childhood experience was positively associated with all fulfillment dimensions, except Positive Impact/Legacy.

**TABLE 13:** Partial correlations between fulfilled life rating, fulfilled life scales, sociodemographic and contextual variables.

	Fulfilled Life	USL	PIL	TWL	FLCE	FLAE
	Rating					
	(retrospect)					
Age	0.20***	0.09*	0.14***	0.14***	0.14***	0.18***
Gender^a^	–0.02	–0.04	0.06	0.05	0.03	0.07
Education	0.10**	0.24***	0.19***	0.09*	0.20***	0.14***
Employment status^b^	–0.02	–0.01	–0.03	–0.04	–0.03	–0.05
Financial status	0.19***	0.20***	0.02	0.11**	0.13***	0.20***
Volunteering^c^	0.03	0.03	0.19***	0.06	0.11**	0.05
Marital status^d^	0.11**	0.07	0.04	0.10**	0.08*	0.12**
Parenthood^e^	0.11**	0.05	0.15***	0.13**	0.13**	0.06
Number of children	0.02	0.02	0.11*	0.06	0.07	0.06
Religion/spirituality	0.07	0.03	0.19***	0.21***	0.17***	0.16***
Self-rated health	0.19***	0.22***	0.12**	0.23***	0.22***	0.27***
Childhood	0.26***	0.24***	0.01	0.20***	0.20***	0.22***

*N_Merged Samples_ = 379–688. All correlations are controlled for age and gender, except age is controlled for gender and gender is controlled for age.*

*USL, Unfolded Self and Life; PIL, Positive Impact and Legacy; TWL, The Worthwhile Life; FLCE, Fulfilled Life Cognitive Experience; FLAE, Fulfilled Life Affective Experience.*

*Childhood was assessed with the question: “How happy was your childhood?”*

*^a^Male = 1, female = 2.*

*^b^0 = employed full- or part-time, self-employed, 1 = retired.*

*^c^0 = no, 1 = yes.*

*^d^0 = single/never married, separated, divorced, widowed, 1 = married, in a registered partnership.*

*^e^0 = no, 1 = yes.*

*p < 0.05; **p < 0.01; ***p < 0.001.

##### A Closer Look at Age

We expected higher fulfillment scores with increasing age and a potential leveling off at the highest age group. Because they consecutively compare an age group with the average of the consecutive groups, Helmert contrasts are well suited to detect such a satiation point. Thus, the merged sample was divided into four groups (covering a decade each), and ANOVAS with subsequent Helmert contrasts were computed for the different fulfillment measures. There was a significant age-group effect on the Unfolded Self and Life, *F*(3,681) = 3.03, *p* = 0.029, η^2^ = 0.01, Helmert contrasts significant at level 1 (*p* = 0.014); Positive Impact Legacy, *F*(3,681) = 6.75, *p* < 0.001, η^2^ = 0.03, Helmert contrasts significant at level 1 (*p* = 0.002) and level 2 (*p* = 0.002); The Worthwhile Life, *F*(3,681) = 5.15, *p* = 0.002, η^2^ = 0.02, Helmert contrasts significant at level 1 (*p* = 0.002) and level 2 (*p* = 0.008); cognitive fulfillment, *F*(3,681) = 6.23, *p* < 0.001, η^2^ = 0.03, Helmert contrasts significant at level 1 (*p* = 0.001) and level 2 (*p* = 0.006); and affective fulfillment, *F*(3,681) = 9.43, *p* < 0.001, η^2^ = 0.04, Helmert contrasts significant at level 1 (*p* < 0.001) and level 2 (*p* = 0.002). Hence, in this cross-sectional study, fulfillment increased gradually with age until the age group of 60–69 years, after which there was no further increase or decrease (i.e., the level of fulfillment stayed the same). The inspection of scatterplots revealed that scores of younger adults ranged between low and high levels, whereas with age, there was a tendency toward higher minimal scores.

## Discussion

The main objective of this study was to develop a reliable and valid measure of a fulfilled life based on our recently proposed theoretical conceptualization (Baumann and Ruch, 2021). The FLS provides an instrument that allows researchers to assess and study the phenomenon of a fulfilled life at different levels. First, it permits to investigate fulfillment at the level of the facets. The assumption that they can be distinguished from each other was supported, as the alpha coefficients were high (even when utilizing only six items); moreover the level of intercorrelation shows that each is characterized by reliable, unique variance. However, the expected pattern of intercorrelations could only be partially substantiated. The three sources (Self, Life, Legacy) and the three criteria (wholeness, fit, value) did not contribute equally and additively to the variance, which eventually led to a revision of the model. The major discovery is that fulfillment regarding the self and one’s life is empirically more intertwined than expected, at least, in terms of the criteria of wholeness and fit. Conceptually, one can postulate a reciprocal relationship between developing the self and having led a fulfilled life, which might have facilitated the intertwining. Both sources are sufficiently different from impact/legacy, where the three criteria intercorrelated so highly that this factor could be extracted. The other surprising deviation is that the value of self and life intercorrelated so highly that they formed a separate factor. Thus, the 3 × 3 bimodal arrangement has been collapsed into a unimodal separation of three components that now form the new structural model: specifically, fulfillment through life and self-actualization, fulfillment through legacy and impact, and fulfillment by experiencing worthwhileness vis a fulfilled life and having fulfilled one’s potential. As the factor structure is replicable, the best items could be selected for the measurement and they yield high Cronbach’s alpha values. The three cognitive components yield a differential correlation pattern with predictors and provide a more nuanced understanding of each subconstruct. Additionally, they may be aggregated and are supplemented by a unidimensional affective component. The high correlation of affective fulfillment with The Worthwhile Life dimension is also noteworthy. It seems in line with theoretical reasoning suggesting that feelings of fulfillment, defined as an affective component, accompany the perception of meaning (Reker and Wong, 1988, 2012). Furthermore, evidence resulting from a multi-method approach has revealed strong associations between positive affect and meaning in life (King et al., 2006). Because the effects can be bidirectional, positive affect serves as a source of information for global life evaluations (e.g., Schwarz, 2001) and, as experimental evidence shows, as an enhancer of meaning, and vice versa, in that the perception of meaning is conducive to positive affect (King et al., 2006). A content-valid rating of fulfillment correlated with all FLS subscales, confirming the validity. Deriving fulfillment from legacy and impact had the comparatively lowest predictive power, and the affective fulfillment scale correlated highest with rated fulfillment. Thus, a valid fulfillment profile can now be studied (at facet and scale levels), which was not feasible before. We also intended to address whether the proposition of a new construct and measure is justified after all, and how the construct is located in its nomological network. In the following sections, we will answer these questions and discuss our findings.

### A Fulfilled Life’s Nomological Network

#### Related Constructs

Regarding convergent and concurrent validity, significant correlations between the FLS and other constructs were achieved. The FLS subscales had small to large correlations with the temporal life satisfaction scales, with the weakest relationships found for Positive Impact and Legacy. This finding implies that the aspect of fulfillment, which includes making a positive difference to others, is scarcely covered by life satisfaction and indicates a limited overlap between the constructs. Depending on the time perspective regarding life satisfaction, the pattern of associations varied. Concerning past life satisfaction, the highest association was found for the Unfolded Self and Life. Hence, favorable past life circumstances might especially impact the extent to which a person would have the ability to realize the self and lead life fully. On the other hand, contributing to others might depend less on excellent living conditions while permitting persons to gain fulfillment regardless of their circumstances. Indeed, a few participants reported deficient levels of retrospective life satisfaction and, at the same time, very high levels of a fulfilled life. Present life satisfaction was most strongly associated with affective fulfillment. In contrast, an examination of future life satisfaction found the highest correlation for the Worthwhile Life, suggesting that looking back on a fulfilled life can inspire hope and confidence.

The small to large strength of the correlations with PERMA suggest that the FLS subscales overlap with some orientations toward well-being yet are distinct. Higher associations were found with an orientation to engagement, accomplishment, and meaning than pleasure and positive relationships. Further results demonstrate that eudaimonic functioning plays a role in attaining a fulfilled life, showing a strong relationship with The Worthwhile Life, in particular. Thus, these findings indicate that FLS covers well-being constructs but is also conceptually and empirically distinct.

Additional evidence for convergent validity came from the finding that a favorable psychosocial development, including attaining generativity and ego integrity, was associated with all fulfilled life dimensions, as was theoretically expected.

Concurrent validity was supported by significant positive correlations between the FLS subscales, mental well-being, self-perceptions of aging, and a calling. Fulfilled individuals reported more positive views of their aging and higher levels of mental well-being. A calling was positively related to all FLS dimensions, and the calling exemplars significantly differed from a general sample. These outcomes confirm the theoretical assumptions that individuals experience a calling as fulfilling. Significantly, a calling is not restricted to age or employment status. In the general sample, retired individuals also yielded high means in a calling.

In summarizing this discussion, the bottom line seems to relate to how fulfilled the sample was in actuality, according to the participants’ responses. The fact that the rating scales were anchored (running from 0 = *not at all fulfilled* to 10 = *entirely fulfilled*) allows that the average fulfillment is 2.5 points away from maximum, and the different age groups are between 3.0 and 1.5 away from being entirely fulfilled (i.e., 10). When rescaling the FLS scales to the same metric, measured fulfillment is roughly one point below rated fulfillment, showing a similar span regarding the age groups. We do not have absolute scales, nevertheless, future studies will produce similar scores if the identical scale is used and can gradually build a reference or a norm to which new studies can compare. Ideally, a representative sample will be collected. When taking the present research in isolation, the majority of people were located above the scale’s midpoint. The question arises whether this can be expected for each sample or whether the current study simply attracted more fulfilled people than a representative sample. Therefore, it will be essential to pay attention to the level of fulfillment in the sample and report it. Eventually, it will also be interesting to compare the mean levels with the means of related constructs (e.g., a particular sample might not be thriving so much but be very fulfilled).

#### Sociodemographic and Contextual Variables

Several results related to sociodemographic variables are worth highlighting, although correlations were small in size. We found age effects for all fulfilled dimensions, with the numerically highest effect on affective fulfillment. This increase in affective fulfillment with age mirrors previous findings on positive emotions across the life span (e.g., Carstensen et al., 2011). The findings revealed an increase in fulfillment until the age of 70, where the level was maintained. Future studies shall take a closer look at high age, employing sufficiently large samples to cover smaller age spans. It seems that over the lifespan, the potential exists to evolve as a person, realize life goals, and make a personally significant contribution. At an advanced age, the reached level of fulfillment might be kept, and people might enjoy the harvest of previous decades.

Further results of this study seem to indicate that education and financial well-being offer persons more opportunities to unfold and lead a life that suits them well. In contrast, we found no associations between financial status and Positive Impact and Legacy. This outcome might imply that persons with fewer financial resources could still make a meaningful contribution and derive fulfillment. Volunteers reported greater fulfillment regarding Positive Impact and Legacy and general cognitive fulfillment than non-volunteers. Through volunteering, individuals gain a sense of mattering and find a meaningful engagement. We found a small positive effect for married in contrast to unmarried persons for The Worthwhile Life, cognitive fulfillment, and affective fulfillment. Marriage seems to provide a sense of significance and meaningfulness (Schnell, 2009), enriching life and contributing to emotional well-being. Parents reported higher levels of fulfillment regarding Positive Impact and Legacy, The Worthwhile Life, and general cognitive fulfillment. Raising children may enable persons to satisfy their need to be generative and allow them to derive meaning and fulfillment by helping their offspring unfold their potential, and become responsible adults. However, this cross-sectional study cannot rule out an alternative causality. Individuals with better subjective health also reported higher levels of fulfillment in all dimensions. This relationship might consist of an interplay between the variables, in that healthier individuals may benefit from greater vitality to engage with life, and the other way around, where fulfilled individuals reap health benefits. Additionally, spirituality is related to all fulfilled life dimensions, except for the Unfolded Self and Life. This finding seems reasonable, as the relation to the transcendent is partly reflected in the Worthwhile Life and the Positive Impact and Legacy dimensions. A positive childhood experience is also related to a fulfilled life, which might imply that a good start is not only critical but may very well pay dividends later. As positive psychology also focuses on enabling positive institutions and communities, it would be worthwhile to help couples maintain successful relationships, strengthen families, and further efforts to promote positive education.

### Proving the Value of This Construct and Measure of a Fulfilled Life

Our findings show that a fulfilled life is a distinct construct and that the FLS is both needed and valuable. To begin with, we found evidence for construct validity. The medium to high correlations between the fulfilled life dimensions and the global ratings demonstrate that the FLS is able to capture participants’ subjective evaluation of a fulfilled life. Next, our study could demonstrate that our scale could predict global fulfillment above existent well-being measures when asking individuals how fulfilled their lives had been. Notably, according to our study findings, a global assessment of a fulfilled life was not sufficiently predicted by hedonic and eudaimonic well-being but required both cognitive and affective fulfillment of the FLS to provide significant increments to the prediction. One reasonable conclusion that can be drawn is that the experience of fulfillment goes beyond hedonic and eudaimonic well-being. These findings also confirm that assessing fulfillment requires both strands in terms of cognitive and affective appraisals. In addition, our measure is necessary to adequately assess the construct. Though the global ratings are economical and can be considered a good criterion for a general orientation, our findings show that they do not cover all aspects of a fulfilled life or do so only insufficiently. The FLS as a multidimensional scale assesses a fulfilled life more differentiated as a global rating, which does not cover the legacy aspect sufficiently. Lastly, our results confirm that the FLS has incremental value beyond established well-being measures. Specifically, the FLS significantly predicted mental well-being above and beyond hedonic and eudaimonic well-being. All these findings justify the importance and additional value of the FLS.

### Implications

Our findings have various implications. A prerequisite for ultimately being able to look back on a fulfilled life might involve reconsidering one’s life periodically and setting the right course. Accordingly, the FLS could be used in psychological counseling on aging or career and life planning, as the assessment helps individuals to reconsider their lives in a differentiated way and reflect on the kind of person they wish to become, the life they want to lead, and the legacy they hope to leave. The result may lead to deeper self-knowledge, greater awareness of what is essential in life, and recognition of various possibilities to shape one’s life. Completing the scale could provide new impulses and inspire persons to be more courageous, particularly addressing the view that individuals might regret missed chances in later life, especially in areas they value most (Roese and Summerville, 2005). The results of the FLS could indicate where to refocus or initiate change. For instance, low scores on the Unfolded Self and Life subscale might suggest that a person should pursue more projects that personally matter, use their strengths to a greater extent, or lead a truer life. The FLS can support people in using their remaining lifetime to live a truly fulfilling life and look back on a life that was well lived according to what they hold most dear.

Further implications pertain to the societal level. Supporting individuals in the second half of life to facilitate living a fulfilling life necessitates revising classic structures in the labor market and education system need revision to permit persons of advanced age to participate in society, realize their potential, find meaningful engagement, and contribute. Appreciation of the gained years due to increasing life expectancy and a more positive view on aging would encourage individuals to consciously use the additional life years and society to capitalize on the human potential of older adults. Therefore, supporting a change in societal attitudes and promoting opportunities for older people would lead to more fulfilled seniors and benefit society as a whole (McNaught, 1994).

### Limitations and Future Research

The studies have several limitations that require some consideration. For example, the samples were convenience samples. Most of the participants were highly educated, and Samples 1 and 2 consisted mainly of women, which may limit the generalizability of the findings. Moreover, as the participants were not paid, we cannot rule out that that some participants volunteered to take part due to their specific interest in the topic. Besides, conducting the study using online surveys could have excluded older individuals, in particular, who were not proficient in using electronic devices. We must point out that while the replication we achieved indicates the stability of a previously found factor structure, it does not provide evidence of model accuracy. Another task for future studies will be establishing test–retest reliability. In this regard, we expect high stability along with an element of malleability. Future studies will also have to continue to explore the role of age. In particular, further research is needed to expand upon the finding that the increase of fulfillment levels off at the age of 70. The high intercorrelation of the subscale The Worthwhile Life with affective fulfillment also needs further investigation. Our studies constitute a first step in empirical research on the promising topic of a fulfilled life. More research is needed to build a more comprehensive understanding of the construct, its correlates, and consequences. For instance, it might be of interest to relate the FLS to the recently proposed concept of psychological richness (Oishi and Westgate, 2021). Alternatively, further research could test the cross-cultural applicability of the FLS. As our scale refers to a fulfilled life in retrospect, future work might construct a scale that assesses a fulfilling life at present or fulfillment in activities. It is necessary to acknowledge that creating a fulfilling life also depends on the environment, including such elements as freedom, security, or institutional quality. Lastly, future research could also examine whether the specific components of a Fulfilled Life could be trained.

## Conclusion

The availability of a reliable and valid measure can pave the way for future research on fulfillment and therefore make an essential contribution to the field of positive psychology and in the area of aging well. The measure holds great practical relevance, too, as it allows individuals to take stock of their lives, gain valuable information, and initiate modifications toward a more fulfilling life. Our results provide first insights into the understanding of a fulfilled life, and further research will be required to achieve a more comprehensive knowledge. Finally, our findings indicate that to arrive at a fulfilled life does count and that a fulfilled life is worth measuring.

## Data Availability Statement

The raw data supporting the conclusions of this article will be made available by the authors, without undue reservation.

## Ethics Statement

Ethical review and approval was not required for the study on human participants in accordance with the local legislation and institutional requirements. The patients/participants provided their written informed consent to participate in this study.

## Author Contributions

DB and WR: conception and design of the work, interpretation of data analysis, and final approval of the published version. DB: data collection, data analysis, and drafting of the manuscript. WR: critical revision of the manuscript. Both authors contributed to the article and approved the submitted version.

## Conflict of Interest

The authors declare that the research was conducted in the absence of any commercial or financial relationships that could be construed as a potential conflict of interest.

## Publisher’s Note

All claims expressed in this article are solely those of the authors and do not necessarily represent those of their affiliated organizations, or those of the publisher, the editors and the reviewers. Any product that may be evaluated in this article, or claim that may be made by its manufacturer, is not guaranteed or endorsed by the publisher.
