# Supplementary material for: Measuring What Counts in Life: The Development and Initial Validation of the Fulfilled Life Scale (FLS)
Source: Front Psychol. 2022 Jan 11;12:795931. doi: 10.3389/fpsyg.2021.795931 (PMC8787361; doi:10.3389/fpsyg.2021.795931)
Supplement: Supplementary file 1 [file Data_Sheet_1.docx]

Supplementary Material

# Table A1 | Item-level descriptive statistics.

|  | *M* | *SD* | *Min* | *Max* | *S* | *K* |
| --- | --- | --- | --- | --- | --- | --- |
| USL |  |  |  |  |  |  |
| 2. I could show my uniqueness. (A) | 4.13 | 1.09 | 1.00 | 6.00 | –0.33 | –0.11 |
| 5. I was able to show my true ability in life. (A) | 4.29 | 1.07 | 1.00 | 6.00 | –0.55 | 0.40 |
| 8. I took advantage of my opportunities in life. (B) | 4.47 | 1.07 | 1.00 | 6.00 | –0.70 | 0.70 |
| 12. I could realize my own dreams. (B) | 4.30 | 1.14 | 1.00 | 6.00 | –0.62 | 0.06 |
| 20. I have had the courage to be as I really am. (D) | 4.39 | 1.21 | 1.00 | 6.00 | –0.62 | 0.01 |
| 21. I have been able to pursue my passions. (D) | 4.33 | 1.08 | 1.00 | 6.00 | –0.48 | 0.14 |
| 26. I could do in life that which I was best suited for. (E) | 4.35 | 1.14 | 1.00 | 6.00 | –0.58 | 0.20 |
| 29. I have led my life in a way that has deeply suited me. (E) | 4.02 | 1.19 | 1.00 | 6.00 | –0.67 | 0.15 |
| PIL |  |  |  |  |  |  |
| 13. I have used opportunities to contribute to others’ well-being. (C) | 4.71 | 1.00 | 1.00 | 6.00 | –0.87 | 1.24 |
| 16. I was able to leave a positive mark with my life on people in my environment. (C) | 4.73 | 0.92 | 1.00 | 6.00 | –0.95 | 1.91 |
| 18. I could make a positive contribution to the other people’s welfare. (C) | 4.74 | 1.01 | 1.00 | 6.00 | –0.99 | 1.59 |
| 34. It was important to me to contribute something to the success of our society. (F) | 4.62 | 1.13 | 1.00 | 6.00 | –0.74 | 0.32 |
| 36. I have used my abilities to make a contribution to the common good. (F) | 4.47 | 1.10 | 1.00 | 6.00 | –0.73 | 0.59 |
| 51. I have significantly supported other people in their development. (I) | 4.79 | 0.99 | 1.00 | 6.00 | –1.01 | 1.84 |
| 52. I could contribute to the success of other people's lives. (I) | 4.68 | 0.99 | 1.00 | 6.00 | –0.66 | 0.63 |
| 54. I have lived for a purpose that goes beyond my life. (I) | 4.01 | 1.35 | 1.00 | 6.00 | –0.41 | -0.58 |
| TWL |  |  |  |  |  |  |
| 39. The efforts in life have been worthwhile. (G) | 4.77 | 1.02 | 1.00 | 6.00 | –0.91 | 1.01 |
| 40. I have the certainty that I have lived for the right things. (G) | 4.50 | 1.05 | 1.00 | 6.00 | –0.76 | 0.84 |
| 43. I have done something valuable with my life. (H) | 4.65 | 1.02 | 1.00 | 6.00 | –0.97 | 1.41 |
| 44. I can look back on a life well lived. (H) | 4.78 | 1.06 | 1.00 | 6.00 | –1.17 | 1.84 |
| 45. Even in the difficult times in life, I have been able to recognize meaning and purpose. (H) | 4.63 | 1.13 | 1.00 | 6.00 | –0.93 | 0.84 |
| 46. My life has been worthwhile. (H) | 4.95 | 1.02 | 1.00 | 6.00 | –1.19 | 1.78 |
| 47. I have experienced my life as meaningful. (H) | 4.80 | 1.02 | 1.00 | 6.00 | –1.22 | 2.27 |
| 48. I have realized what really matters in life. (H) | 4.62 | 1.07 | 1.00 | 6.00 | –0.91 | 1.28 |
| FLAE |  |  |  |  |  |  |
| 1. I feel deep inner contentment. | 4.33 | 1.20 | 1.00 | 6.00 | –0.89 | 0.59 |
| 2. I feel in harmony with myself and the lived life. | 4.49 | 1.13 | 1.00 | 6.00 | –0.90 | 0.66 |
| 3. I have inner peace. | 4.44 | 1.09 | 1.00 | 6.00 | –0.85 | 0.90 |
| 5. I feel great gratitude. | 4.95 | 1.18 | 1.00 | 6.00 | –1.26 | 1.46 |
| 6. I feel fulfilled. | 4.44 | 1.16 | 1.00 | 6.00 | –0.91 | 0.64 |
| 7. I feel deep regret. (R) | 5.14 | 1.14 | 1.00 | 6.00 | –1.41 | 1.41 |
| 11. I feel disappointed. (R) | 4.97 | 1.22 | 1.00 | 6.00 | –1.26 | 1.10 |
| 12. I feel rather empty. (R) | 5.08 | 1.19 | 1.00 | 6.00 | –1.37 | 1.32 |

*Note.* Sample 2 = *N*_Replication_ = 406. Scale range: 1-6. *S* = Skewness. *K* = Kurtosis. Reverse-scored items are denoted with (R). English items were translated from the original German items employing a translation/backtranslation procedure. The translation has not been validated.

The Fulfilled Life Scale (FLS) and Scoring Information

FLCE

The following statements refer to your *life lived so far in retrospect*. Please rate how well each of these statements applies to you personally. Try to answer as honestly as possible. There are no right or wrong answers.

1. I could show my uniqueness.
2. The efforts in life have been worthwhile.
3. I have used opportunities to contribute to others’ well-being.
4. I was able to show my true ability in life.
5. I have the certainty that I have lived for the right things.
6. I was able to leave a positive mark with my life on people in my environment.
7. I took advantage of my opportunities in life.
8. I have done something valuable with my life.
9. I could make a positive contribution to other people’s welfare.
10. I could realize my own dreams.
11. I can look back on a life well lived.
12. It was important to me to contribute something to the success of our society.
13. I have had the courage to be as I really am.
14. Even in the difficult times in life, I have been able to recognize meaning and purpose.
15. I have used my abilities to make a contribution to the common good.
16. I have been able to pursue my passions.
17. My life has been worthwhile.
18. I have significantly supported other people in their development.
19. I could do in life that which I was best suited for.
20. I have experienced my life as meaningful.
21. I could contribute to the success of other people’s lives.
22. I have led my life in a way that has deeply suited me.
23. I have realized what really matters in life.
24. I have lived for a purpose that goes beyond my life.

FLAE

The following statements also relate to your life lived so far. What feelings and sensations do you have when you look back on the life you have lived so far? Please rate how well each of these statements applies to you personally.

**When I look back on my life, ...**

1. I feel deep inner contentment.
2. I feel deep regret.
3. I feel rather empty.
4. I feel in harmony with myself and the lived life.
5. I feel great gratitude.
6. I have inner peace.
7. I feel fulfilled.
8. I feel disappointed.

Scoring

Responses are rated on a 6-point scale (*does not apply at all, does not apply, rather does not apply, rather applies, applies, applies completely*). The assignment of the items to the subscales is presented below. False-keyed items are denoted by “R”. Subscale scores are computed by averaging item responses in each subscale. The scale mean can range from a minimum of 1 to a maximum of 6 points. To compute the total score of the Fulfilled Life Cognitive Experience (FLCE), compute the grand mean of all three subscale means.

1. Unfolded Self and Life: 1, 4, 7, 10, 13, 16, 19, 22
2. The Worthwhile Life: 2, 5, 8, 11, 14, 17, 20, 23
3. Positive Impact and Legacy: 3, 6, 9, 12, 15, 18, 21, 24

Fulfilled Life Affective Experience (FLAE): 1, 2R, 3R, 4, 5, 6, 7, 8R

Citation

Baumann, D., & Ruch, W. (2021). Measuring what counts in life: The development and initial validation of the fulfilled life scale (FLS). *Frontiers in Psychology, 12:* 795931. https://doi.org/10.3389/fpsyg.2021.795931
